# Polyadenine insertion disrupting the *G6PC1* gene in German Pinschers with glycogen storage disease type Ia (GSD1A)

**DOI:** 10.1111/age.13146

**Published:** 2021-10-05

**Authors:** Matthias Christen, Wencke Reineking, Andreas Beineke, Vidhya Jagannathan, Wolfgang Baumgärtner, Tosso Leeb

**Affiliations:** ^1^ Institute of Genetics, Vetsuisse Faculty University of Bern Bern 3001 Switzerland; ^2^ Department of Pathology University of Veterinary Medicine Hannover 30559 Germany

## Background

Glycogen storage diseases (GSD) are a group of inherited disorders of carbohydrate metabolism that occur in humans and animals. Variants in 20 different genes are currently known to result in GSD in humans.^1^ One of the candidate genes is *G6PC1* encoding glucose‐6‐phosphatase catalytic subunit 1. *G6PC1* loss‐of‐function variants cause GSD1A, which is also termed von Gierke disease. The disease is characterized by severe hypoglycemia and hepatomegaly owing to hepatic glycogen accumulation early in life.^2^ A canine form of GSD1A has been identified in Maltese Terriers and is caused by a p.Met121Ile variant in the *G6PC1* gene (OMIA 000418‐9615).^3^ Dogs with GSD1A have become a popular, naturally occurring animal model for gene‐therapy research in this disease.^1997^, ^2001^, ^2018^

## Analyses

Two 4‐week‐old purebred German Pinscher puppies, one male and one female, showed an enlarged abdomen at clinical examination and poor weight gain since birth. Ultrasonography of the female puppy revealed an increased liver size. Furthermore, this puppy was reported to be bilaterally blind. The male puppy developed seizures. The affected puppies were euthanized owing to worsening of clinical signs and poor prognosis. At necropsy, both dogs showed severe hepatomegaly with light brown to yellow discoloration and fragile consistency of the liver. Histologically, diffuse swelling and vacuolization of hepatocytes with peripheral displacement of nuclei was found. Periodic acid Schiff‐positive, diastase‐sensitive material was present in hepatocytes, indicating glycogen accumulation.

We isolated genomic DNA from both cases from liver tissue samples and sequenced the genome of one affected dog at approximately 18.6× coverage with Illumina 2 × 150 bp reads. The data were analyzed as described previously^6^ and submitted to the European Nucleotide Archive under sample accession no. SAMEA8157169. Filtering for variants present only in the sequenced dog and absent in 795 control genomes (Table␣S1) yielded no homozygous private protein‐changing variant in a known GSD candidate gene^1^ (Table␣S2).

We then visually inspected the short read alignments in the candidate genes for structural variants using the Integrative Genomics Viewer.^7^ This revealed a homozygous 76 bp insertion into exon 5 of the *G6PC1* candidate gene, which probably causes a loss of function (chr9:g.20,134,857_20,134,858ins76; XM_038676372.1:c.634_635ins76). The insertion consisted of 60 consecutive adenines and an additional 16 bp duplication of the integration site (Figure␣1, File S1). The variant was confirmed by Sanger sequencing and an additional 208 unrelated German Pinscher dogs were genotyped. Only the two affected dogs carried the insertion in a homozygous state. However, 24 additional heterozygous carriers were identified corresponding to a carrier frequency of 12%.

**Figure 1:**
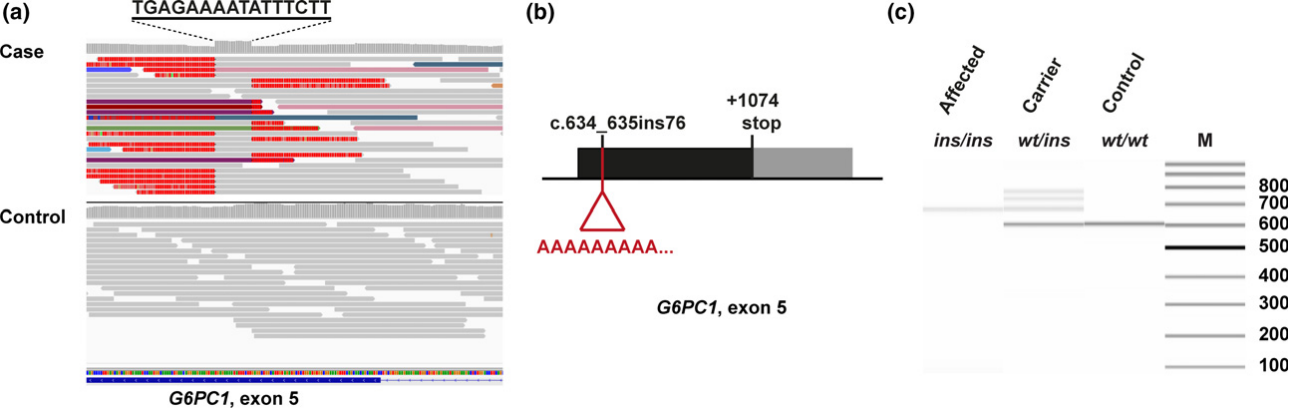
Insertion into exon 5 of the *G6PC1* gene. (a) Integrative Genomics Viewer screenshot illustrating the structural variant in the orientation of the CanFam 3.1 genome reference sequence. The case shows an increased coverage over 16 nucleotides characteristic for the duplication of sequences at transposable element integration sites spanning Chr9:20,134,842–20,134,857 (CanFam3.1 assembly). Soft‐clipped bases deviating from the genome reference to the left and the right side of this duplication appear bright red owing to the presence of poly‐T stretches. Colored reads indicate that their mates map to other chromosomes. These features are characteristic of an insertion of a repetitive element into the genome of the affected dog. (b) Schematic representation of the insertion into the last exon of the *G6PC1* gene. As *G6PC1* is in reverse complementary orientation with respect to the genome reference, the insertion is now represented as a poly‐A stretch. (c) Fragment size analysis of the PCR amplification products obtained from genomic DNA with primers 5′‐GCATGCAATGGGAGAATGTA‐3′ and 5′‐AGGTGCAGGAGGATGAGAGA‐3′. Representative results of an affected dog (*ins/ins*), heterozygous carrier (*wt/ins*) and a healthy control (*wt/wt*) are shown. The samples were analyzed on a 5200 FragmentAnalyzer capillary gel electrophoresis instrument (Agilent). In the carrier dog, the two minor bands that migrated more slowly than the two main products most likely represent heteroduplex molecules consisting of one mutant and one wt strand.

## Conclusions

Based on the clinical and histological findings GSD was diagnosed in two German Pinscher puppies. A precision medicine approach identified a 76 bp SINE insertion in *G6PC1* as the most likely candidate causative variant and allowed designation of the phenotype as GSD1A. The disease allele is present at non‐negligible frequency in the German Pinscher dog population and the introduction of genetic testing for breeding animals is recommended.

## Supporting information


**Table␣S1**. Accession numbers of 787 dog and nine wolf genome sequences.

**Table␣S2**. Private variants in the sequenced dog affected with glycogen storage disease.

**File S1**. Sequence context of the 76 bp A‐rich insertion into exon 5 of the canine *G6PC1* gene.
