# Supplementary material for: Polyadenine insertion disrupting the G6PC1 gene in German Pinschers with glycogen storage disease type Ia (GSD1A)
Source: Anim Genet. 2021 Oct 5;52(6):900–2. doi: 10.1111/age.13146 (PMC9293233; doi:10.1111/age.13146)
Supplement: Supplementary file 3 — File S1. Sequence context of the 76 bp A‐rich insertion into exon 5 of the canine G6PC1 gene. [file AGE-52-900-s003.pdf]

File S1. Sequence context of the 76 bp A-rich insertion into exon 5 of the canine *G6PC1* gene

Genetic variant:	chr9:g.20,134,857_20,134,858ins76	(CanFam 3.1 assembly)
	G6PC1:c.634_635ins76	(XM_038676372.1)

CanFam 3.1, chromosome 9 genomic sequence, positions 20,134,401-20,135,300 reverse complement

Wildtype *G6PC1* exon 5 in black CAPITAL LETTERS, intronic bases in small letters, 76 bp insertion in blue

PCR primers for the amplification of a genomic 601 bp product (wildtype allele) vs. a genomic 677 bp product (variant allele) are indicated in green and beneath the sequence with “>>>” and “<<<”.

```
20,135,300     tagaggggga cacatttcag cttagcacaa ggaagaattt tttataatca aatccattca     20,135,241
20,135,240     aacatgaaat gggctgcccg agtgggagtg agctctctac ctctgcaagt attcaagtag     20,135,181
20,135,180     aaacataagg acccccaaca ggaaacatgc atgcaatggg agaatgtatg ggacatgact     20,135,121
                                            >>>>>>>>>>>>>>>>>>>>>>
20,135,120     cttccatctg aaagagtgta tgactaagga cagattggag tcacatagga gaataaacca     20,135,061
20,135,060     ggtgactctc taatctagca tctgtgattt aatgccacaa tggcaggatg gaccatatgt     20,135,001
20,135,000     tacctattgc tccaaacact ccccacctct gcggctgaag gtccagagtc ttccctattt     20,135,941
20,134,940     ctcacagcca ttctttcttc cacgcagGCA TTGCTGTTGC TGAAACTTTC CGCCACATCC     20,134,881
20,134,880     AGAGCATCTA CAATGCCAGC CTCAAGAAAT ATTTTCTCA                            20,134,142
                                       16 nt duplication flanking the insertion

20,134,143.76  AAAAAAAAAA AAAAAAAAAA AAAAAAAAAA AAAAAAAAAA AAAAAAAAAA AAAAAAAAAA     20,134,143.17
20,134,143.16  AAGAAATATT TTCTCA                                                    20,134,143.1
               16 nt duplication flanking the insertion

20,134,143                                        T TACTTTCTTC CTGTTCAGTT     20,134,821
20,134,820     TTGCCATTGG ATTTTACCTG CTGCTCAAGG GGCTGGGTGT GGACCTCCTG TGGACACTGG     20,134,761
20,134,760     AAAAAGCCAG GAGATGGTGT GAGCGGCCGG AATGGGTTCA CATTGACACC ACACCCTTTG     20,134,701
20,134,700     CCAGCCTTCT CAAGAACGTG GGGACCCTCT TTGGCCTGGG GGTGGCTCTC AACTCCAGCA     20,134,641
20,134,640     TGTACAGGGA AAGCTGCAAG GGCAAGCTTA GCAAGTGGTT CCCATTCCGC CTCAGCTGCA     20,134,581
20,134,580     TTGTGGTGTC TCTCATCCTC CTGCACCTCT TTGACTCTTT GAAACCCCCA TCCCAAACTG     20,134,521
                       <<<<<<<<<<<<<<<<<<<<<<
20,134,520     AGCTGATCTT CTACACCTTG TCCTTCTGCA AGAGTGCAGC AGTGCCCCTG GCATCTGTCA     20,134,461
20,134,460     GCCTCATCCC CTACTGCCTT GCCCGGGTCT TCGACCAGCC AGACAAGAAG TCTTTGTAAG     20,134,401
                                                                  stop codon
```
